# Supplementary material for: Burden of Traumatic Brain Injuries in Children and Adolescents in Europe: Hospital Discharges, Deaths and Years of Life Lost
Source: Children (Basel). 2022 Jan 13;9(1):105. doi: 10.3390/children9010105 (PMC8775116; doi:10.3390/children9010105)
Supplement: Supplementary file 1 [file children-09-00105-s001.zip › Figure S1.pdf]

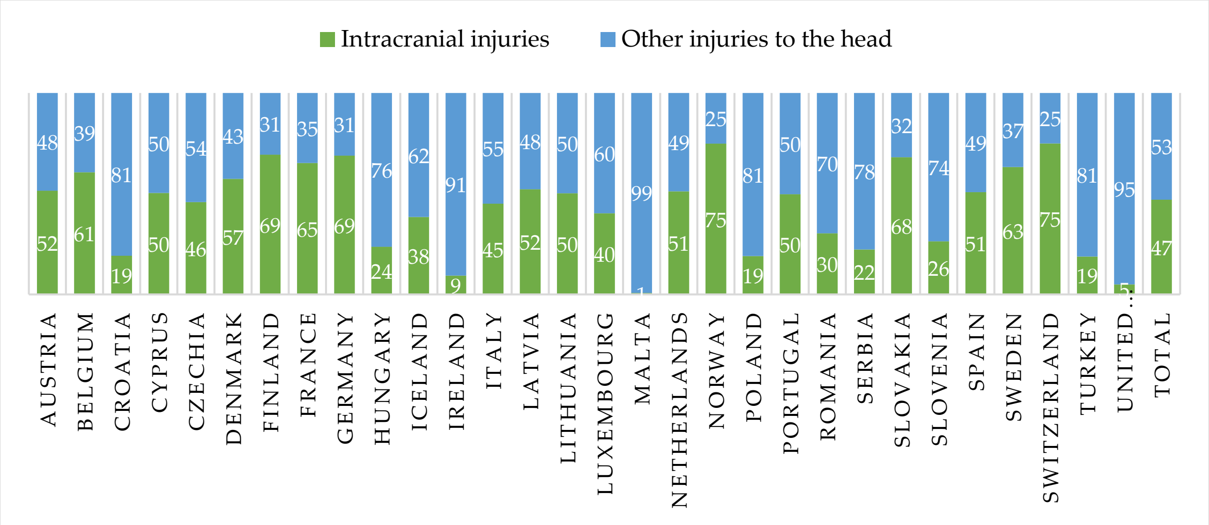

Figure S1. Proportions of intracranial injuries (ISHMT 1901) and other head injuries (ISHMT 1902) in 0–19 years old in 30 European countries by country, both sexes combined, in 2014.

ISHMT: International Shortlist for Hospital Morbidity Tabulation.
