# Supplementary material for: Burden of Traumatic Brain Injuries in Children and Adolescents in Europe: Hospital Discharges, Deaths and Years of Life Lost
Source: Children (Basel). 2022 Jan 13;9(1):105. doi: 10.3390/children9010105 (PMC8775116; doi:10.3390/children9010105)
Supplement: Supplementary file 1 [file children-09-00105-s001.zip › Figure S2.pdf]

A

Country	Rate (Cr)	Rate (Adj)	95% CI (Adj)
Germany	1019.2	1019.9	(1012.7–1027.2)
Austria	984.5	972.9	(952.3–993.8)
Czech Republic	763.4	758.1	(741.5–775.0)
Poland	645.7	639.6	(631.9–647.5)
Latvia	619.6	628.8	(594.0–665.1)
Switzerland	606.8	599.5	(583.2–616.1)
Malta	589.7	584.1	(515.3–659.9)
Slovenia	585.9	574.9	(542.6–608.7)
Slovakia	515.0	508.0	(489.9–526.7)
Litva	493.5	484.1	(460.0–509.1)
Cyprus	467.9	457.0	(416.3–500.7)
Ireland	453.8	445.2	(429.2–461.6)
Belgium	448.3	440.9	(429.6–452.5)
Hungary	430.7	433.8	(421.0–446.9)
United Kingdom	410.2	398.5	(394.1–402.9)
France	391.5	387.9	(383.7–392.2)
Croatia	375.8	374.1	(356.4–392.4)
Romania	328.6	328.5	(320.9–336.3)
Norway	325.1	324.0	(310.3–338.2)
Sweden	306.2	301.6	(291.6–311.9)
Serbia	282.4	281.5	(269.4–294.0)
Netherlands	254.4	254.7	(247.7–261.9)
Finland	242.4	240.7	(228.6–253.2)
Denmark	208.5	208.4	(197.7–219.6)
Italy	173.0	172.4	(169.0–175.8)
Turkey	148.2	147.4	(145.3–149.5)
Luxembourg	132.5	132.1	(105.5–163.5)
Spain	107.1	106.3	(103.3–109.2)
Iceland	79.0	79.5	(55.6–110.3)
Portugal	43.2	61.8	(57.3–66.6)
**Pooled**	**414.4**	**411.0**	**(337.0–486.0)**

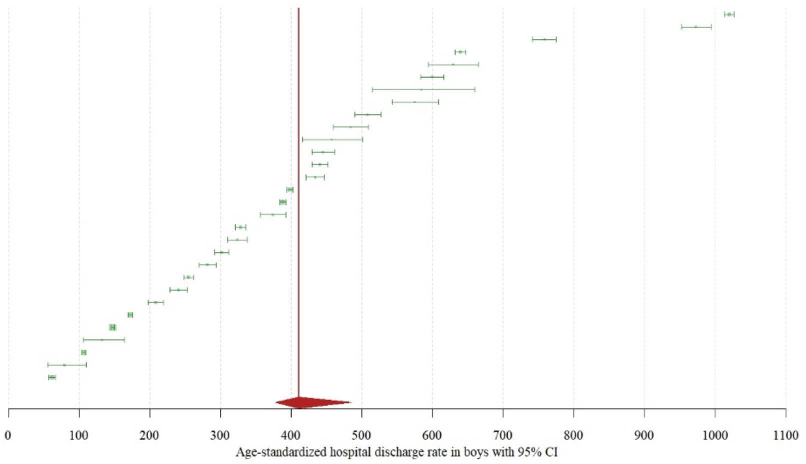

B

Country	Rate (Cr)	Rate (Adj)	95% CI (Adj)
Lithuania	12.2	11.4	(8.0–15.7)
Bulgaria	8.6	8.8	(6.7–11.3)
Cyprus	8.0	7.3	(3.1–14.7)
Finland	7.1	7.0	(5.1–9.4)
Greece	6.6	6.6	(5.2–8.3)
Croatia	6.7	6.3	(4.3–9.1)
Luxembourg	6.2	6.2	(1.7–15.9)
Estonia	5.8	6.1	(2.6–11.2)
Austria	4.8	4.6	(3.3–6.2)
Turkey	4.3	4.2	(3.9–4.6)
Czech Republic	3.8	4.1	(2.9–5.5)
Malta	4.5	3.9	(0.5–15.6)
Latvia	3.5	3.7	(1.5–7.7)
Switzerland	3.5	3.5	(2.3–4.9)
Romania	3.5	3.5	(2.7–4.1)
Serbia	2.8	2.7	(1.6–4.2)
Belgium	2.3	2.4	(1.6–3.4)
Iceland	2.2	2.2	(0.1–12.9)
Norway	2.2	2.2	(1.2–3.6)
Denmark	2.2	2.1	(1.2–3.5)
Netherlands	2.1	2.1	(1.5–2.8)
Portugal	1.4	2.1	(1.3–3.1)
Slovenia	2.0	2.1	(0.6–5.3)
Germany	2.0	1.9	(1.6–2.2)
Italy	1.9	1.9	(1.6–2.3)
United Kingdom	1.9	1.9	(1.6–2.3)
Sweden	1.9	1.8	(1.2–2.8)
Slovakia	2.0	1.8	(0.9–3.3)
Ireland	1.6	1.7	(0.9–3.1)
Hungary	1.5	1.4	(0.8–2.4)
**Pooled**	**4.0**	**3.9**	**(3.3–4.6)**

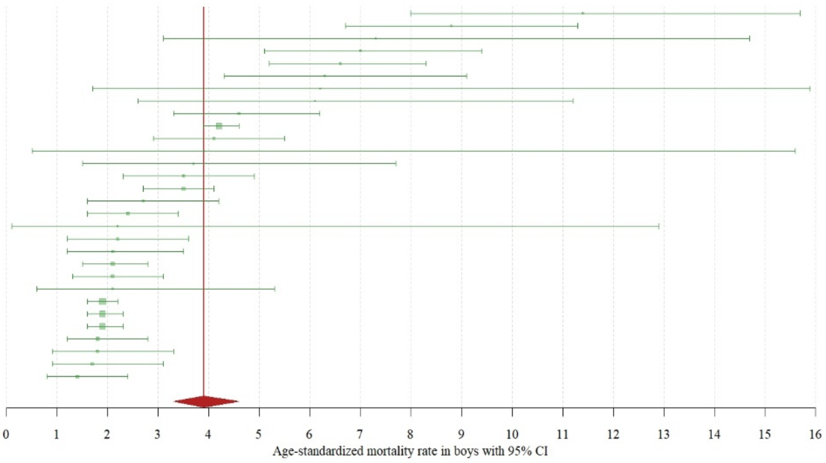

C

Country	Rate (Cr)	Rate (Adj)	95% CI (Adj)
Lithuania	712.2	665.2	(637.7–693.7)
Bulgaria	509.3	517.6	(500.4–535.2)
Finland	471.6	464.0	(447.3–481.2)
Cyprus	510.0	462.2	(422.9–504.4)
Greece	423.3	425.2	(413.1–437.7)
Luxembourg	391.4	386.0	(339.7–436.9)
Croatia	405.7	385.7	(368.1–403.9)
Estonia	351.2	367.3	(335.2–401.8)
Austria	311.1	293.4	(282.4–304.7)
Turkey	280.7	278.4	(275.6–281.3)
Czech Republic	243.0	252.9	(243.1–262.9)
Malta	285.9	250.2	(208.6–298.3)
Switzerland	237.6	232.6	(222.6–243.0)
Romania	216.0	215.5	(209.3–221.8)
Latvia	193.7	204.2	(184.3–225.7)
Serbia	164.8	160.2	(151.2–169.6)
Belgium	151.5	153.6	(146.9–160.6)
Norway	147.3	146.2	(137.0–155.8)
Iceland	140.5	142.1	(109.4–181.7)
Denmark	143.7	140.1	(131.4–149.2)
Netherlands	138.8	138.2	(131.1–143.5)
Portugal	89.0	131.7	(125.1–138.6)
Italy	127.2	127.6	(124.7–130.6)
United Kingdom	127.4	127.2	(124.7–129.7)
Slovenia	120.2	126.4	(111.1–143.3)
Sweden	123.3	124.0	(117.5–130.7)
Germany	127.0	121.3	(118.9–123.8)
Ireland	114.9	118.3	(110.0–127.1)
Slovakia	113.9	109.4	(101.2–118.1)
Hungary	88.7	86.0	(80.5–91.8)
**Pooled**	**248.7**	**245.1**	**(198.2–291.9)**

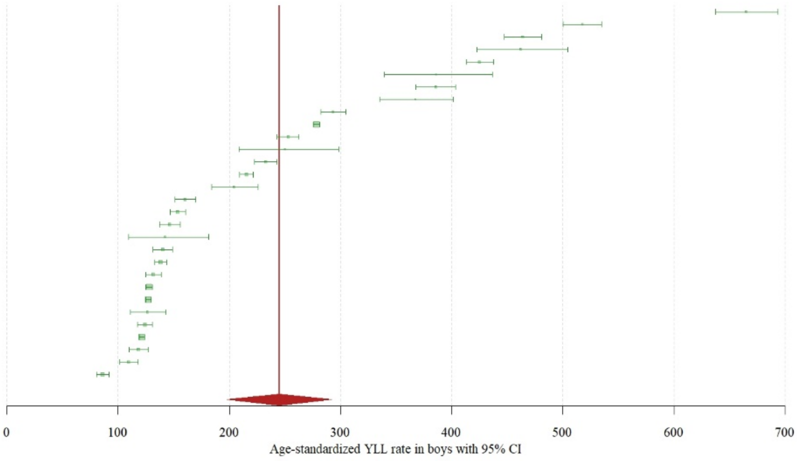

Figure S2. Crude and age-standardized rates of TBI-related hospital discharges (A), deaths (B) and YLLs (C) with estimated pooled rates for the analyzed 30 European countries in 2014, boys.
