# Supplementary material for: Burden of Traumatic Brain Injuries in Children and Adolescents in Europe: Hospital Discharges, Deaths and Years of Life Lost
Source: Children (Basel). 2022 Jan 13;9(1):105. doi: 10.3390/children9010105 (PMC8775116; doi:10.3390/children9010105)
Supplement: Supplementary file 1 [file children-09-00105-s001.zip › Figure S3.pdf]

**A**

| Country | Rate (Cr) | Rate (Adj) | 95% CI (Adj) |
|---|---|---|---|
| Germany | 824.2 | 823.2 | (816.5–829.9) |
| Austria | 732.6 | 726.3 | (708.0–744.9) |
| Czech Republic | 553.4 | 539.8 | (525.5–554.3) |
| Switzerland | 477.2 | 468.9 | (454.2–484.0) |
| Poland | 416.2 | 411.7 | (405.3–418.1) |
| Latvia | 393.6 | 393.5 | (365.6–423.1) |
| Slovenia | 391.8 | 373.8 | (347.5–401.7) |
| Malta | 359.6 | 352.3 | (298.0–414.1) |
| Slovakia | 343.9 | 336.9 | (321.9–352.5) |
| Hungary | 327.8 | 329.3 | (317.9–341.0) |
| Belgium | 310.4 | 301.5 | (292.0–311.2) |
| Lithuania | 301.8 | 301.1 | (281.5–321.6) |
| Cyprus | 272.6 | 266.4 | (234.7–301.2) |
| Ireland | 269.4 | 250.7 | (238.8–263.1) |
| Croatia | 242.1 | 241.4 | (226.9–256.7) |
| France | 241.1 | 235.4 | (232.1–238.7) |
| Sweden | 236.8 | 231.4 | (222.4–240.7) |
| United Kingdom | 241.0 | 229.2 | (225.8–232.6) |
| Norway | 229.9 | 227.9 | (216.1–240.1) |
| Netherlands | 188.8 | 189.3 | (183.1–195.6) |
| Romania | 165.3 | 165.6 | (160.1–171.3) |
| Finland | 158.7 | 156.8 | (146.9–167.1) |
| Denmark | 144.8 | 145.1 | (135.9–154.7) |
| Serbia | 143.7 | 143.8 | (135.0–153.1) |
| Italy | 94.1 | 93.0 | (90.5–95.5) |
| Luxembourg | 80.5 | 79.9 | (59.1–105.8) |
| Turkey | 67.5 | 66.6 | (65.1–68.0) |
| Spain | 57.2 | 55.3 | (53.2–57.5) |
| Portugal | 36.8 | 37.3 | (33.6–41.3) |
| Iceland | 36.6 | 36.3 | (20.7–59.2) |
| **Pooled** | **278.0** | **273.6** | **(206.5–340.8)** |

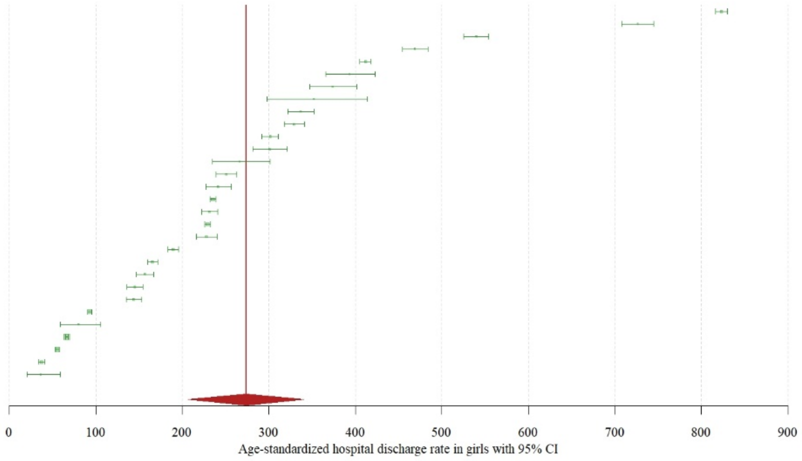


**B**

| Country | Rate (Cr) | Rate (Adj) | 95% CI (Adj) |
|---|---|---|---|
| Lithuania | 6.7 | 6.4 | (3.9–10.0) |
| Estonia | 4.6 | 5.1 | (1.8–11.2) |
| Bulgaria | 3.1 | 3.1 | (1.8–4.8) |
| Switzerland | 3.2 | 3.1 | (2.0–4.6) |
| Slovenia | 2.8 | 2.8 | (0.1–7.4) |
| Cyprus | 2.1 | 2.3 | (0.3–8.3) |
| Iceland | 2.3 | 2.3 | (0.1–13.3) |
| Croatia | 2.4 | 2.2 | (1.0–4.2) |
| Malta | 2.4 | 2.1 | (0.1–13.6) |
| Finland | 2.0 | 2.0 | (1.0–3.5) |
| Turkey | 1.9 | 1.8 | (1.6–2.1) |
| Greece | 1.7 | 1.7 | (1.0–2.6) |
| Latvia | 1.6 | 1.5 | (0.3–4.6) |
| Romania | 1.5 | 1.5 | (0.9–2.1) |
| United Kingdom | 1.1 | 1.1 | (0.9–1.4) |
| Hungary | 1.0 | 1.0 | (0.5–1.9) |
| Serbia | 1.0 | 1.0 | (0.4–2.1) |
| Sweden | 1.0 | 1.0 | (0.5–1.8) |
| Slovakia | 1.0 | 1.0 | (0.4–2.3) |
| Austria | 1.0 | 0.9 | (0.4–1.8) |
| Denmark | 0.9 | 0.9 | (0.3–1.9) |
| Netherlands | 0.9 | 0.9 | (0.5–1.4) |
| Belgium | 0.8 | 0.8 | (0.4–1.5) |
| Czech Republic | 0.9 | 0.8 | (0.4–1.7) |
| Germany | 0.8 | 0.8 | (0.6–1.0) |
| Portugal | 0.8 | 0.8 | (0.3–1.5) |
| Italy | 0.7 | 0.7 | (0.5–0.9) |
| Norway | 0.6 | 0.6 | (0.2–1.7) |
| Ireland | 0.5 | 0.5 | (0.1–1.6) |
| Luxembourg | 0.0 | 0.0 | (0.0–0.0) |
| **Pooled** | **1.7** | **1.7** | **(1.3–2.1)** |

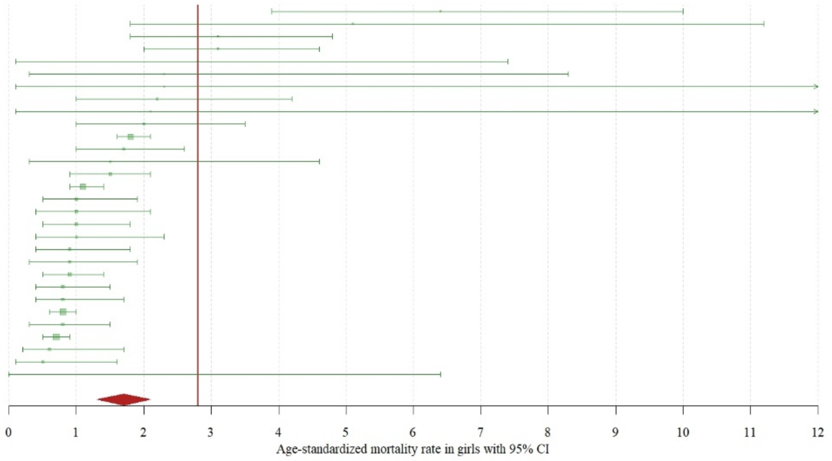


**C**

| Country | Rate (Cr) | Rate (Adj) | 95% CI (Adj) |
|---|---|---|---|
| Lithuania | 460.4 | 439.0 | (415.9–463.1) |
| Estonia | 314.6 | 353.3 | (320.0–389.1) |
| Switzerland | 240.9 | 233.9 | (223.6–244.6) |
| Bulgaria | 216.6 | 211.9 | (200.8–223.4) |
| Cyprus | 163.8 | 178.3 | (151.3–208.7) |
| Slovenia | 155.3 | 165.7 | (147.4–185.6) |
| Iceland | 155.6 | 159.2 | (123.7–202.0) |
| Croatia | 163.5 | 156.6 | (145.1–168.7) |
| Finland | 152.1 | 149.6 | (140.0–159.8) |
| Malta | 163.0 | 145.7 | (113.1–185.4) |
| Turkey | 134.3 | 132.2 | (130.2–134.3) |
| Greece | 131.1 | 128.8 | (122.1–135.8) |
| Latvia | 118.6 | 113.8 | (99.3–129.8) |
| Romania | 103.1 | 103.2 | (98.8–107.7) |
| United Kingdom | 82.0 | 80.6 | (78.6–82.6) |
| Sweden | 73.0 | 73.5 | (68.4–78.9) |
| Hungary | 72.8 | 71.8 | (66.5–77.3) |
| Slovakia | 74.2 | 71.7 | (64.9–79.0) |
| Serbia | 65.9 | 64.8 | (59.0–71.1) |
| Austria | 66.8 | 63.6 | (58.3–69.1) |
| Netherlands | 63.7 | 63.1 | (59.6–66.8) |
| Czech Republic | 65.0 | 63.0 | (58.3–68.1) |
| Denmark | 64.5 | 62.9 | (57.0–69.2) |
| Germany | 60.2 | 58.2 | (59.5–60.0) |
| Belgium | 56.8 | 57.6 | (53.4–62.1) |
| Portugal | 57.1 | 56.3 | (51.8–61.1) |
| Italy | 55.0 | 54.6 | (52.7–56.6) |
| Norway | 45.8 | 46.5 | (41.2–52.3) |
| Ireland | 32.7 | 37.6 | (32.6–43.1) |
| Luxembourg | 0.0 | 0.0 | (0.0–0.0) |
| **Pooled** | **120.3** | **119.9** | **(91.8–148.0)** |

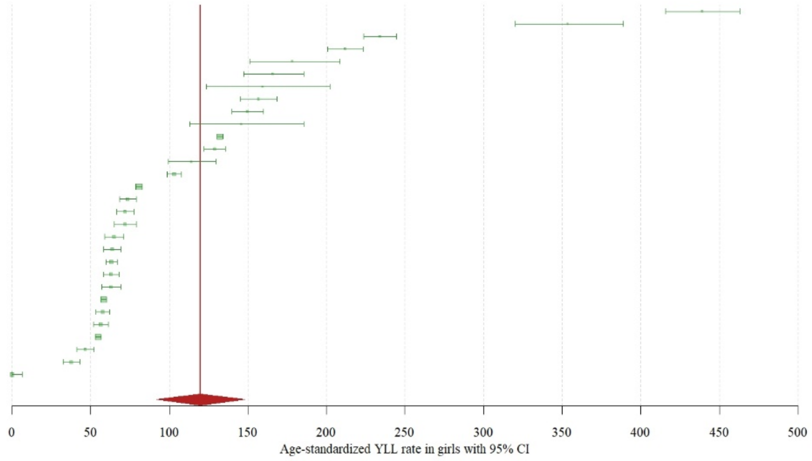


**Figure S3.** Crude and age-standardized rates of TBI-related hospital discharges (A), deaths (B) and YLLs (C) with estimated pooled rates for the analyzed 30 European countries in 2014, girls.
