# Supplementary material for: Burden of Traumatic Brain Injuries in Children and Adolescents in Europe: Hospital Discharges, Deaths and Years of Life Lost
Source: Children (Basel). 2022 Jan 13;9(1):105. doi: 10.3390/children9010105 (PMC8775116; doi:10.3390/children9010105)
Supplement: Supplementary file 1 [file children-09-00105-s001.zip › Figure S5.pdf]

A

Country	Rate (Cr)	Rate (Adj)	95% CI (Adj)
Germany	970.2	971.7	(964.7–978.8)
Austria	892.1	883.3	(863.6–903.2)
Czech Republic	751.2	745.9	(729.4–762.6)
Latvia	598.9	607.6	(573.4–643.3)
Switzerland	606.8	599.5	(583.2–616.1)
Malta	560.5	556.5	(489.3–630.7)
Slovenia	564.9	554.4	(522.7–587.6)
Slovakia	505.5	498.7	(480.8–517.1)
Lithuania	479.1	470.0	(446.3–494.7)
Hungary	429.6	432.8	(420.0–445.9)
Poland	395.3	392.6	(386.5–398.8)
Cyprus	396.7	388.6	(351.1–429.1)
Ireland	394.9	382.9	(368.2–398.0)
Belgium	330.7	324.0	(314.3–333.9)
Croatia	298.8	298.1	(282.3–314.6)
France	262.8	260.0	(256.6–263.5)
Serbia	260.1	259.3	(247.7–271.3)
Romania	227.7	227.5	(221.2–234.0)
Netherlands	226.1	226.5	(219.9–233.3)
Norway	222.2	221.8	(210.5–233.7)
Sweden	223.6	221.1	(212.6–230.0)
Finland	175.1	174.0	(163.7–184.7)
United Kingdom	170.2	166.1	(163.2–168.9)
Italy	162.5	161.7	(158.5–165.0)
Turkey	148.2	147.4	(145.3–149.5)
Denmark	101.2	101.1	(93.7–109.0)
Luxembourg	98.2	97.7	(75.0–125.1)
Spain	90.7	90.2	(87.5–93.0)
Iceland	79.0	79.5	(55.6–110.3)
Portugal	38.6	55.5	(51.3–60.1)
**Pooled**	**355.4**	**353.2**	**(232.7–473.7)**

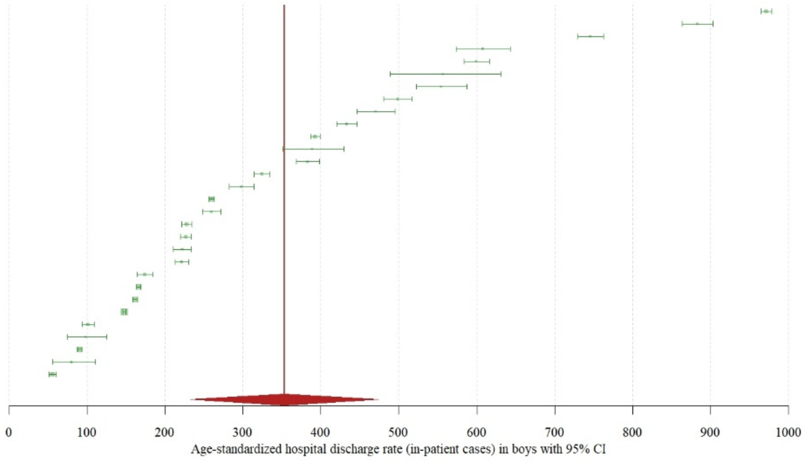

B

Country	Rate (Cr)	Rate (Adj)	95% CI (Adj)
Poland	250.4	247.0	(242.2–251.9)
United Kingdom	240.0	232.4	(229.1–235.8)
France	128.7	127.9	(125.5–130.3)
Belgium	117.6	116.9	(111.1–123.0)
Denmark	107.3	107.3	(99.7–115.4)
Norway	102.9	102.2	(94.5–110.3)
Romania	100.9	101.0	(96.8–105.4)
Austria	92.4	89.6	(83.5–96.1)
Sweden	82.6	80.5	(75.4–85.8)
Croatia	77.0	76.0	(68.1–84.4)
Cyprus	71.1	68.4	(53.4–86.5)
Finland	67.4	66.7	(60.5–73.5)
Ireland	58.9	62.3	(56.2–68.9)
Germany	49.0	48.2	(46.6–49.8)
Luxembourg	34.3	34.4	(21.5–52.2)
Netherlands	28.3	28.2	(25.9–30.6)
Malta	29.3	27.6	(14.6–47.8)
Serbia	22.4	22.2	(18.9–25.9)
Latvia	20.7	21.2	(15.2–28.9)
Slovenia	21.0	20.5	(14.8–27.7)
Spain	16.4	16.0	(14.9–17.2)
Lithuania	14.8	14.3	(10.5–19.2)
Czech Republic	12.2	12.2	(10.2–14.5)
Italy	10.6	10.6	(9.8–11.5)
Slovakia	9.5	9.3	(7.0–12.2)
Portugal	4.6	6.3	(4.9–7.9)
Hungary	1.1	1.1	(0.5–1.9)
**Pooled**	**65.6**	**64.8**	**(52.3–77.3)**

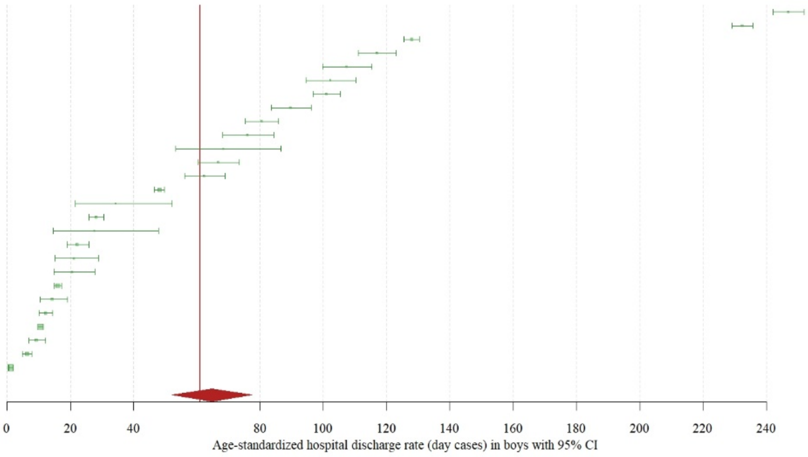

Figure S5: Crude and age-standardized rates of TBI-related in-patient (A) and day cases (B) hospital discharges with estimated pooled rates for the analysed 30 European countries in 2014, in boys.
