# Supplementary material for: Burden of Traumatic Brain Injuries in Children and Adolescents in Europe: Hospital Discharges, Deaths and Years of Life Lost
Source: Children (Basel). 2022 Jan 13;9(1):105. doi: 10.3390/children9010105 (PMC8775116; doi:10.3390/children9010105)
Supplement: Supplementary file 1 [file children-09-00105-s001.zip › Figure S6.pdf]

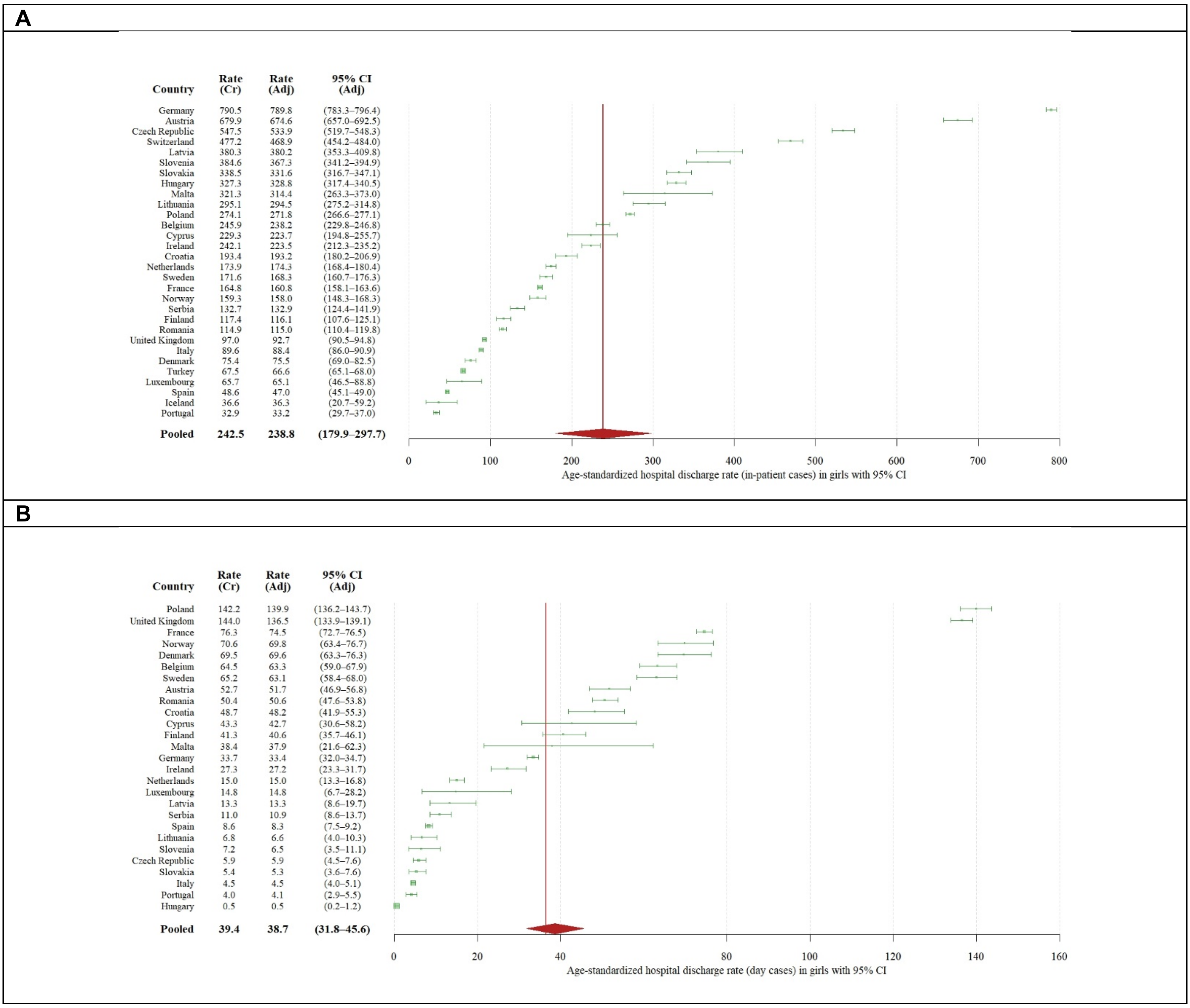

Figure S6: Crude and age-standardized rates of TBI-related in-patient (A) and day cases (B) hospital discharges with estimated pooled rates for the analysed 30 European countries in 2014, in girls.
