# Supplementary material for: Burden of Traumatic Brain Injuries in Children and Adolescents in Europe: Hospital Discharges, Deaths and Years of Life Lost
Source: Children (Basel). 2022 Jan 13;9(1):105. doi: 10.3390/children9010105 (PMC8775116; doi:10.3390/children9010105)
Supplement: Supplementary file 1 [file children-09-00105-s001.zip › Table S1.pdf]

Table S1. Case definitions of TBI related deaths, external causes of deaths and hospital discharges due to TBI.

**ICD-10 codes used to define TBI in the causes of death dataset**		**ICD-10 codes used to define external causes of injury in fatal TBI**	
**S00**	**Superficial injury of head**	V01–V99	Transport accidents
S01	Open wound of head	W00–W19	Falls
S02	Fracture of skull and facial bones	X60–X84	Intentional self-harm
S03	Dislocation and sprain of joints and ligaments of head	X85–Y09	Assault
S04	Injury of cranial nerve	Y10–Y89	Other
S05	Injury of eye and orbit		
S06	Intracranial injury	**IHMT categories used to define TBI in hospital discharges**	
S07	Crushing injury of head	ISHMT 1901	Intracranial injuries, includes ICD-10 code S06
S08	Avulsion and traumatic amputation of part of head		
S09	Other and unspecified injuries of head	ISHMT 1902	Other head injuries, includes ICD-10 codes S00–S05 and S07–S09
T90	Sequelae of injuries of head		

ISHMT: International Shortlist for Hospital Morbidity Tabulation.
