# Supplementary material for: Burden of Traumatic Brain Injuries in Children and Adolescents in Europe: Hospital Discharges, Deaths and Years of Life Lost
Source: Children (Basel). 2022 Jan 13;9(1):105. doi: 10.3390/children9010105 (PMC8775116; doi:10.3390/children9010105)
Supplement: Supplementary file 1 [file children-09-00105-s001.zip › Table S2.pdf]

Table S2. Availability of the data on hospital discharges and deaths due to TBI per country.

Country (Total 33)	Hospital discharges	Hospital discharges including separate data on day-cases and in-patient data)	Deaths
**Austria**	Yes	Yes	Yes
**Belgium**	Yes	Yes	Yes
**Bulgaria**	Not available	Not available	Yes
**Croatia**	Yes	Yes	Yes
**Cyprus**	Yes	Yes	Yes
**Czech Republic**	Yes	Yes	Yes
**Denmark**	Yes	Yes	Yes
**Estonia**	Not available	Not available	Yes
**Finland**	Yes	Yes	Yes
**France**	Yes	Yes	Not available
**Germany**	Yes	Yes	Yes
**Greece**	Not available	Not available	Yes
**Hungary**	Yes	Yes	Yes
**Iceland**	Yes	Not available	Yes
**Ireland**	Yes	Yes	Yes
**Italy**	Yes	Yes	Yes
**Latvia**	Yes	Yes	Yes
**Lithuania**	Yes	Yes	Yes
**Luxembourg**	Yes	Yes	Yes
**Malta**	Yes	Yes	Yes
**Netherlands**	Yes	Yes	Yes
**Norway**	Yes	Yes	Yes
**Poland**	Yes	Yes	Not available
**Portugal**	Yes	Yes	Yes
**Romania**	Yes	Yes	Yes
**Serbia**	Yes	Yes	Yes
**Slovakia**	Yes	Yes	Yes
**Slovenia**	Yes	Yes	Yes
**Spain**	Yes	Yes	Not available
**Sweden**	Yes	Yes	Yes
**Switzerland**	Yes	Not available	Yes
**Turkey**	Yes	Not available	Yes
**United Kingdom**	Yes	Yes	Yes
**Total of countries with data available**	**30**	**27**	**30**
