# Supplementary material for: Burden of Traumatic Brain Injuries in Children and Adolescents in Europe: Hospital Discharges, Deaths and Years of Life Lost
Source: Children (Basel). 2022 Jan 13;9(1):105. doi: 10.3390/children9010105 (PMC8775116; doi:10.3390/children9010105)
Supplement: Supplementary file 1 [file children-09-00105-s001.zip › Table S3.pdf]

Table S3. Sex ratios of hospital discharges, deaths and YLLs by country.

Country	Hospital discharges	Deaths	Years of life lost
**Austria**	1.3 (1.3–1.4)	3.9 (2.9–5.4)	4.6 (4.2–5.0)
**Belgium**	1.4 (1.4–1.5)	2.5 (1.8–3.4)	2.6 (2.4–2.9)
**Bulgaria**	not available	2.6 (2.0–3.3)	2.3 (2.2–2.5)
**Croatia**	1.5 (1.4–1.7)	3.0 (2.1–4.5)	2.5 (2.2–2.7)
**Cyprus**	1.7 (1.5–2.0)	2.3 (1.0–5.6)	3.1 (2.6–3.7)
**Czech Republic**	1.4 (1.3–1.4)	2.9 (2.0–4.2)	3.7 (3.4–4.0)
**Denmark**	1.4 (1.3–1.5)	2.6 (1.4–5.0)	2.2 (2.0–2.5)
**Estonia**	not available	4.8 (2.7–9.4)	1.1 (1.0–1.3)
**Finland**	1.5 (1.4–1.6)	2.5 (1.8–3.5)	3.0 (2.8–3.3)
**France**	1.6 (1.6–1.6)	not available	not available
**Germany**	1.2 (1.2–1.2)	2.3 (2.0–2.7)	2.1 (2.0–2.2)
**Greece**	not available	3.3 (2.5–4.4)	3.2 (3.0–3.4)
**Hungary**	1.3 (1.2– 1.4)	2.3 (1.6–3.5)	1.2 (1.1–1.3)
**Iceland**	2.1 (1.2–3.9)	0.9 (0.2–5.4)	0.9 (0.6–1.2)
**Ireland**	1.7 (1.6–1.8)	4.2 (2.3–8.5)	3.5 (3.0–4.0)
**Italy**	1.8 (1.8–1.9)	2.2 (1.8–2.6)	2.3 (2.2–2.4)
**Latvia**	1.6 (1.4–1.7)	2.2 (1.4–3.8)	1.6 (1.4–1.9)
**Lithuania**	1.6 (1.5–1.7)	2.5 (1.9–3.4)	1.5 (1.4–1.6)
**Luxembourg**	1.6 (1.1–2.3)	1.8 (0.7–5.4)	–
**Malta**	1.6 (1.3–2.0)	0.9 (0.1–9.0)	1.7 (1.3–2.4)
**Netherlands**	1.3 (1.3–1.4)	1.7 (1.3–2.3)	2.1 (2.0–2.3)
**Norway**	1.4 (1.3–1.5)	1.9 (1.1–3.4)	3.2 (2.8–3.6)
**Poland**	1.5 (1.5–1.6)	not available	not available
**Portugal**	1.9 (1.7–2.2)	3.5 (2.2–5.8)	2.6 (2.3–2.8)
**Romania**	2.0 (1.9–2.0)	2.4 (2.0–2.8)	2.1 (2.0–2.2)
**Serbia**	2.0 (1.8–2.1)	4.0 (2.4–7.1)	2.5 (2.2–2.8)
**Slovakia**	1.5 (1.4–1.6)	1.8 (1.2–2.8)	1.5 (1.3–1.7)
**Slovenia**	1.5 (1.4–1.6)	1.9 (0.9–3.9)	0.8 (0.7–0.9)
**Spain**	1.9 (1.8–2.0)	not available	not available
**Sweden**	1.3 (1.2–1.3)	1.5 (1.0–2.2)	1.7 (1.5–1.8)
**Switzerland**	1.3 (1.2–1.3)	1.9 (1.4–2.6)	1.0 (0.9–1.0)
**Turkey**	2.2 (2.1–2.2)	2.3 (2.2–2.5)	2.0 (1.9–2.0)
**United Kingdom**	1.7 (1.6–1.7)	2.3 (2.0–2.6)	1.5 (1.5–1.6)
**Average**	**2.4 (2.3–2.5)**	**1.5 (1.5–1.5)**	**2.0 (2.0–2.0)**
