# Supplementary material for: Burden of Traumatic Brain Injuries in Children and Adolescents in Europe: Hospital Discharges, Deaths and Years of Life Lost
Source: Children (Basel). 2022 Jan 13;9(1):105. doi: 10.3390/children9010105 (PMC8775116; doi:10.3390/children9010105)
Supplement: Supplementary file 1 [file children-09-00105-s001.zip › Table S4.pdf]

Table S4. Proportions (%) of external causes of death due to TBI in 0–19 years old in 30 European countries by country, both sexes combined, in 2014.

Countries	Traffic	Falls	Suicide	Violence	Other
**Austria**	68	0	16	4	12
**Belgium**	55	13	10	8	15
**Bulgaria**	44	21	8	5	23
**Croatia**	80	0	0	20	0
**Cyprus**	62	4	12	0	22
**Czech Republic**	70	5	12	5	8
**Denmark**	57	14	24	5	0
**Estonia**	71	14	14	0	0
**Finland**	50	4	21	14	11
**Germany**	71	7	7	2	13
**Greece**	65	10	15	10	0
**Hungary**	72	4	0	0	24
**Italy**	64	11	4	9	13
**Ireland**	93	0	0	0	7
**Iceland**	100	0	0	0	0
**Latvia**	74	6	9	3	9
**Lithuania**	69	7	0	3	21
**Luxembourg**	100	0	0	0	0
**Malta**	70	0	0	10	20
**Netherlands**	74	0	12	3	10
**Norway**	33	33	33	0	0
**Portugal**	39	17	17	11	17
**Romania**	73	6	9	0	12
**Serbia**	72	8	0	3	17
**Slovakia**	78	4	0	4	15
**Slovenia**	53	6	28	6	6
**Sweden**	25	0	25	25	25
**Switzerland**	59	6	6	0	29
**Turkey**	56	7	5	3	29
**United Kingdom**	67	4	3	3	23
**Total**	**63**	**7**	**7**	**4**	**19**

See Supplementary Table S1 for definitions of external cause groups.
