# Supplementary material for: Burden of Traumatic Brain Injuries in Children and Adolescents in Europe: Hospital Discharges, Deaths and Years of Life Lost
Source: Children (Basel). 2022 Jan 13;9(1):105. doi: 10.3390/children9010105 (PMC8775116; doi:10.3390/children9010105)
Supplement: Supplementary file 1 [file children-09-00105-s001.zip › Table S5.pdf]

Table S5. Estimated numbers of hospital discharges, deaths and YLLs due to TBI extrapolated to the population of 0–19 years old in the European Union for 2014, by sex.

	Pooled crude rate (95% CI)	Estimated number of cases (95% CI)
	**Boys**	
**Hospital discharges due to TBI – overall**	414.4 (338.0–491.0)	226,279 (184,561–268,105)
**Hospital discharges – intracranial injuries**	177.7 (138.7–216.7)	97,031 (75,736–118,327)
**Hospital discharges – other head injuries**	236.7 (186.5–286.9)	129,248 (101,836–156,659)
**Deaths due to TBI**	4.0 (3.3–4.6)	2184 (1802–2512)
**YLLs due to TBI**	248.7 (198.5–298.9)	135,800 (108,389–163,211)
	**Girls**	
**Hospital discharges due to TBI – overall**	278.0 (209.8–346.2)	144,110 (108,757–179,464)
**Hospital discharges – intracranial injuries**	133.3 (107.1–159.5)	69,100 (55,519–82,682)
**Hospital discharges – other head injuries**	144.7 (115.4–173.9)	75,010 (59,821–90,147)
**Deaths due to TBI**	1.7 (1.3–2.1)	881 (674–1,089)
**YLLs due to TBI**	120.3 (92.1–148.4)	62,361 (47,743–76,928)

For extrapolation, the population estimate of 0–19 years old in the European Union for 2014 was used (population count: boys 54,603,941, girls 51,838,271).
